# Unilateral choroidal detachment in an elderly patient with Vogt-Koyanagi-Harada disease: a case report and literature review

**DOI:** 10.3389/fimmu.2025.1514306

**Published:** 2025-02-27

**Authors:** Chuzhi Peng, Yonghong Jiao, Chunli Chen

**Affiliations:** ^1^ Beijing Tongren Eye Center, Beijing Tongren Hospital, Capital Medical University, Beijing, China; ^2^ Beijing Key Laboratory of Ophthalmology and Visual Sciences, Beijing, China

**Keywords:** Vogt-Koyanagi-Harada disease, choroidal detachment, serous retinal detachment, multimodal imaging, VKH disease

## Abstract

**Purpose:**

To report an uncommon case of Vogt-Koyanagi-Harada (VKH) disease in an elderly patient with unilateral choroidal detachment and describe its multimodal imaging features and prognosis.

**Method:**

Case report and literature review of clinical features in VKH with choroidal detachment.

**Results:**

A 76-year-old woman presented with bilateral blurred vision and headache 6 months prior to visiting our hospital. She was diagnosed with iridocyclitis at another hospital and received local anti-inflammatory treatment without improvement. Slit-lamp examination showed bilateral mutton-fat and dust-like keratic precipitates, anterior chamber and vitreous cells, and posterior synechiae in the right eye. Fundus examination revealed bilateral optic disc swelling and choroidal detachment in the left eye. Fluorescein angiography revealed bilateral optic disc leakage, punctate hyperfluorescence in the posterior pole, and elevated fluorescence leakage in the left eye’s temporal area. Indocyanine Green Angiography showed multiple of choroidal hypoperfusion areas in the left eye, with an elevated fluorescence blockage on the temporal side. Optical coherence tomography showed subretinal fluid, wavy retinal pigment epithelium, and choroidal thickening in both eyes. Based on ocular and neurological findings, the patient was diagnosed with bilateral VKH. After ruling out infectious factors, she received high-dose systemic corticosteroids and immunosuppressants. The choroidal detachment and serous retinal detachment gradually resolved.

**Conclusion:**

This case is the first report of unilateral choroidal detachment associated with VKH in an elderly patient. VKH patients with choroidal detachment reported in previous studies were predominantly elderly and Asian, characterized by optic disc hyperfluorescence and choroidal detachment. Multimodal imaging can help clinicians better diagnose and manage atypical types of VKH.

## Introduction

Vogt-Koyanagi-Harada (VKH) disease is an autoimmune disorder characterized by chronic granulomatous uveitis in both eyes, often accompanied by changes in neurological system, inner ear, and skin. It is more common in certain pigmented races, particularly Asian, Middle Eastern, Hispanic, and Native American (1–3). The most common age for onset of VKH is between 20 and 50 years (2, 4), but children (5) and elderly individuals (6) can also be affected. Women are more likely to develop the disease than men (7, 8). Immunopathological studies indicated that VKH is associated with autoimmune inflammation mediated by CD4+ T cells targeting melanocytes (9). Tyrosinase peptides probably acts as the autoantigen in VKH, with T cells T cells mounting an immune response by targeting the tyrosinase peptides produced by melanocytes (10). In addition, the human leukocyte antigen (HLA) genotype (DRB1*0405) is closely associated with genetic susceptibility to VKH (11, 12). The ocular features of VKH mainly include pan-uveitis with serous retinal detachment, and in the late stage, sunset glow fundus may appear (3). Systemic manifestations in the prodromal phase include headache, fever, neck stiffness, vertigo, tinnitus, and hearing loss. In later stages of the disease, vitiligo and poliosis may occur (7). Here, we report a case of an elderly VKH patient with unilateral choroidal detachment and bilateral serous retinal detachment. Furthermore, we summarize and review the clinical features of previously reported cases of choroidal detachment in VKH.

## Case report

**Figure 1:**
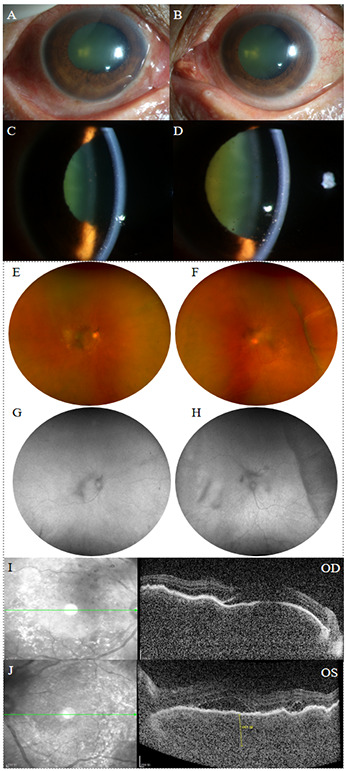
Anterior segment photography, fundus photography, fundus autofluorescence, and OCT images of the patient at her initial visit to our hospital. **(A-D)** Both eyes showed ciliary injection, a slightly shallow anterior chamber, a large amount of mutton-fat and dust-like keratic precipitates and anterior chamber cells (+++). The right eye showed iris posterior synechiae. **(E, F)** Fundus photography showed bilateral mild optic disc swelling and blurred disc margins, with retinal folds and scattered yellow-white lesions in the posterior pole. Choroidal detachment was observed in the temporal region of the left eye. **(G, H)** Fundus autofluorescence showed low autofluorescence in the vitreous of both eyes, with a hypoautofluorescent elevated area in the temporal region of the left eye. **(I, J)** OCT revealed wavy RPE, subretinal fluid, and significant choroidal thickening in both eyes. The subretinal fluid in the left eye appeared compartmentalized.

**Figure 2:**
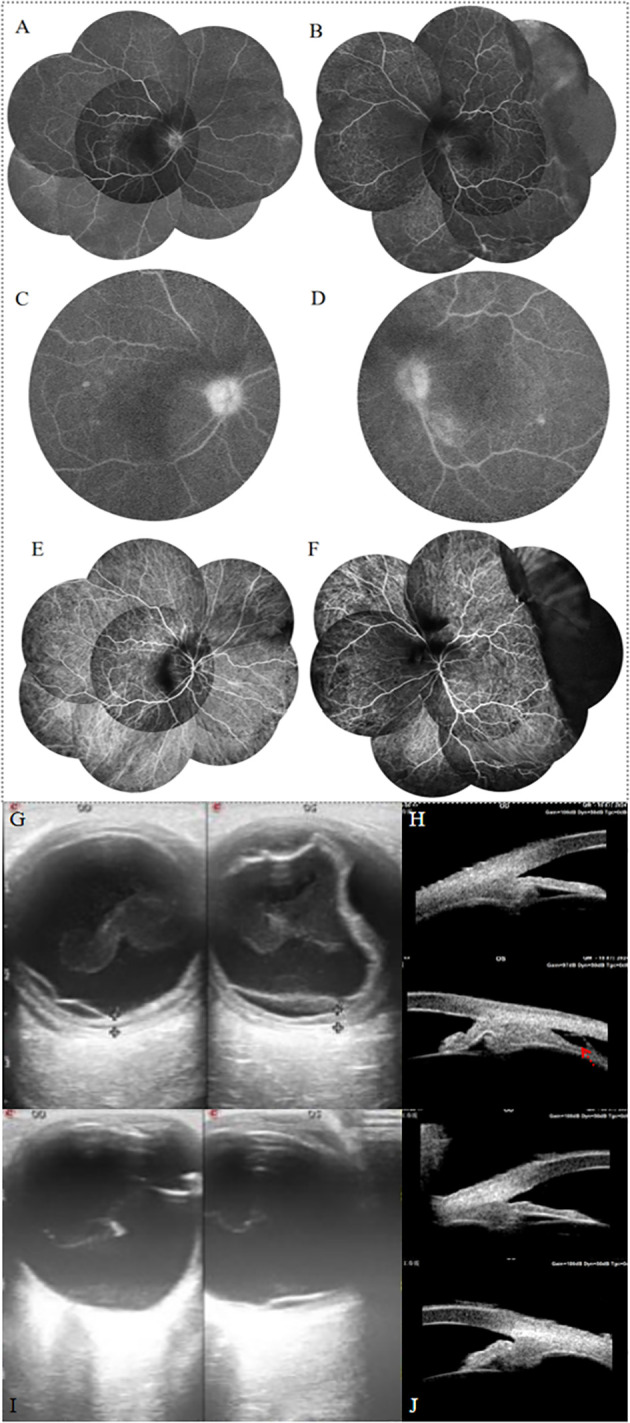
**(A, B)** FFA revealed peripheral retinal vessel wall staining of both eyes, with blurred disc margins and hyperfluorescence of the optic disc. The left eye showed numerous punctate and linear areas of hypofluorescence, with retinal elevation in the temporal and inferior peripheral regions accompanied by vascular wall staining. **(C, D)** Late-phase FFA showed optic disc leakage in both eyes, with punctate-patchy hypofluorescence in the posterior pole. **(E, F)** Indocyanine green angiography showed numerous punctate and linear hypofluorescence in both eyes. In the left eye, multiple areas of choroidal hypoperfusion are observed, with an elevated fluorescence blockage on the temporal side. **(G)** Ocular ultrasonography revealed weak punctate and continuous band-like echoes in the vitreous of both eyes, and diffuse thickening of the ocular wall echo in the right eye. In the left eye, one end of the echo was attached to the peripheral ocular wall, while the other end was connected to the equatorial ocular wall, with an anechoic area beneath. Color Doppler Flow Imaging detected blood flow signals along the band-like echoes in both eyes. Retinal detachment was observed in both eyes, along with choroidal detachment in the left eye. No associated scleral thickening, masses, or “T” sign was noted. **(H)** UBM revealed abnormal anterior segment echoes in both eyes, with anterior synechiae of the iris and suprachoroidal effusion in the left eye (red arrow). After 2 weeks of treatment, Ocular ultrasonography showed significant recovery of the choroidal detachment in the left eye **(I)**, and UBM revealed abnormal anterior segment echoes in both eyes and the resolution of suprachoroidal effusion in the left eye **(J)**.

**Figure 3:**
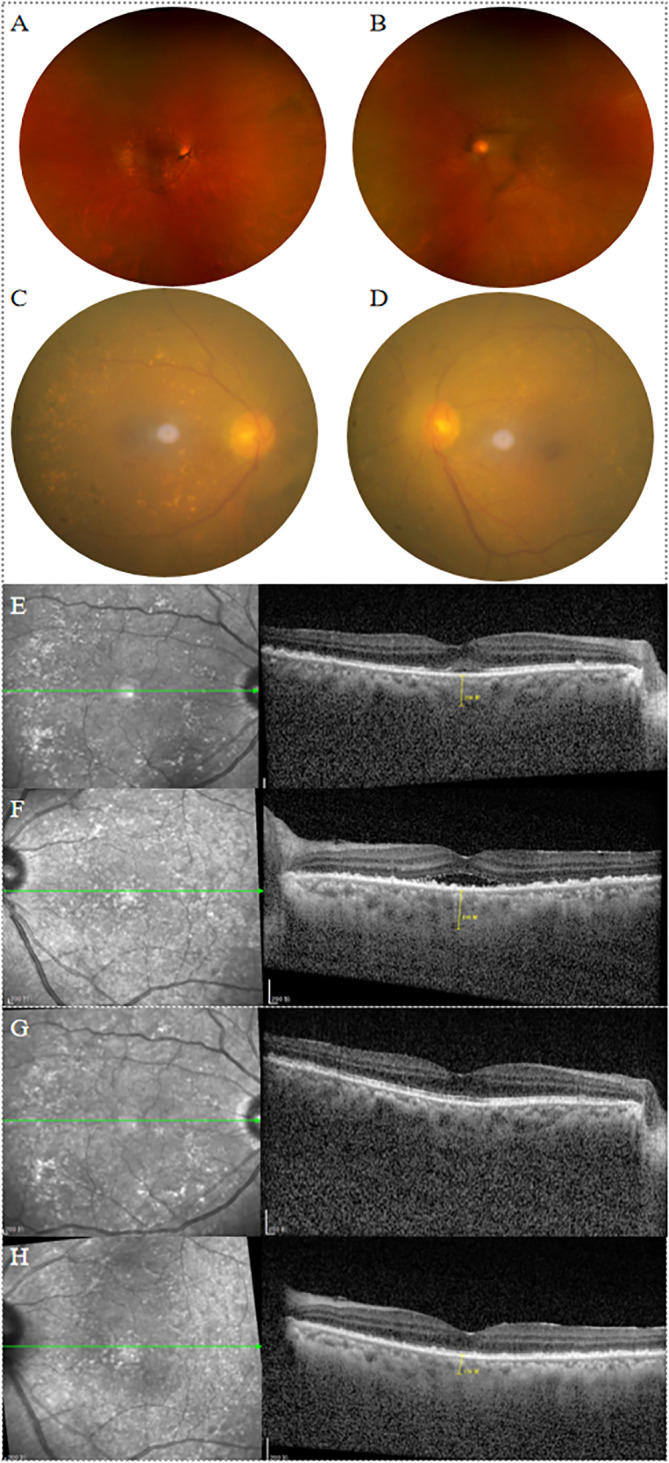
**(A-D)** After 2 weeks of treatment, fundus images showed vitreous opacity, a reddish optic disc with clear margins, dilated veins, and flat retina in both eyes. Numerous yellow-white lesions were observed in the posterior pole of both eyes, and choroidal detachment in the left eye had resolved. **(E, F)** After 2 weeks of treatment, OCT showed significant absorption of subretinal fluid in both eyes, with a marked decrease in choroidal thickness. **(G, H)** After one and a half months of treatment, OCT showed resolution of the serous retinal detachment in both eyes, with choroidal thickness returning to normal. In the right eye, the ellipsoid zone in the macular area appeared thinned, while the left eye showed disruption and loss of the ellipsoid zone, accompanied by high-reflective signals.

A 76-year-old woman presented with progressive bilateral blurred vision for 6 months. Prior to the significant vision loss, she sought consultation at a local hospital’ s neurology department due to headache, dizziness, and neck stiffness. The local ophthalmologist diagnosed her with bilateral iridocyclitis and treated her with non-steroidal anti-inflammatory and steroid eye drops. However, her symptoms did not improve, and vision progressively deteriorated. Her medial and ocular history were unremarkable and the patient denied oral ulcers, joint pain, or skin rash. At the first visit to our hospital, her visual acuity was 0.1 in the right eye and counting fingers (with no improvement upon correction) in the left. The intraocular pressure was 14 mmHg in the right eye and 12 mmHg in the left. The axial length was 23.61 mm in the right eye and 23.37 mm in the left. The scleral thickness was 0.48mm in the right eye and 0.46mm in the left. Slit-lamp examination revealed bilateral ciliary injection, a slightly shallow anterior chamber, a large number of mutton-fat and dust-like keratic precipitates, anterior chamber cells (+++), Tyndall effect (+++), and lens opacity (**Figures 1A–D**). The right eye also showed iris posterior synechiae. Apparent vitreous opacity with a large number of inflammatory cells were detected. Fundus examination showed bilateral mild optic disc swelling and blurred disc margins. The cup-to-disc ratio was small, with retinal folds and scattered yellow-white lesions visible in the posterior pole. Choroidal detachment was observed in the temporal region of the left eye (**Figures 1E, F**). Fundus autofluorescence examination showed low autofluorescence in the vitreous of both eyes, with a hypoautofluorescent elevated area visible in the temporal region of the left eye (**Figures 1G, H**). Optical coherence tomography (OCT) revealed wavy retinal pigment epithelium (RPE), subretinal fluid, and significant choroidal thickening in both eyes. The subretinal fluid in the left eye appeared compartmentalized (**Figures 1I, J**). Fundus fluorescein angiography (FFA) in the venous phase showed retinal vein staining of both eyes, with blurred disc margins and hyperfluorescence of the optic disc (**Figures 2A–D**). Punctate and patchy hyperfluorescence was observed in the posterior pole of the right eye. The left eye showed numerous punctate and linear areas of hypofluorescence, with retinal elevation in the temporal and inferior peripheral regions accompanied by vascular wall staining. Severe hyperfluorescent leakage of the optic disc was observed in the late stage. Indocyanine green angiography showed numerous punctate hypofluorescent areas in all quadrants of the retina in both eyes during both early and late phases (**Figures 2E, F**). Linear hypofluorescence was observed in the left eye, with fluorescent blockage in the temporal peripheral region. Ocular ultrasonography revealed weak punctate and continuous band-like echoes in the vitreous of both eyes, and diffuse thickening of the ocular wall echo in the right eye. In the left eye, one end of the echo was attached to the peripheral ocular wall, while the other end was connected to the equatorial ocular wall, with an anechoic area beneath. Color Doppler Flow Imaging detected blood flow signals along the band-like echoes in both eyes. Retinal detachment was observed in both eyes, along with choroidal detachment in the left eye. No associated scleral thickening, masses, or “T” sign was noted (**Figure 2G**). Ultrasound biomicroscopy (UBM) revealed abnormal anterior segment echoes in both eyes, with anterior synechiae of the iris and suprachoroidal effusion in the left eye (**Figure 2H**). The results of the complete blood count, C-reactive protein, erythrocyte sedimentation rate, rheumatoid factor, serology for syphilis, HIV antibody test, hepatitis B virus antigen, hepatitis C virus antigen, serum angiotensin converting enzyme, chest CT, and T-spot test were all within normal limits. The patient’s HLA typing was negative. The patient was diagnosed with bilateral complete VKH based on her ocular abnormalities and extra-ocular symptoms (13). The patient initially received a posterior subtenon injection of 20 mg triamcinolone acetonide in both eyes. After ruling out infectious causes, the patient was started on oral prednisone (1.0 mg/kg) with gradual tapering. Concurrently, the patient began treatment with oral methotrexate, and topical treatments with prednisolone acetate, pranoprofen, and compound tropicamide eye drops. Two weeks later, the choroidal detachment in the left eye and retinal detachment in the right eye completely resolved. The serous retinal detachment in the left eye significantly subsided, and the choroidal thickness in both eyes markedly decreased (**Figures 3A–F**). Ocular ultrasonography showed resolution of the choroidal detachment in the left eye (**Figure 2I**), and UBM revealed abnormal anterior segment echoes in both eyes and the resolution of suprachoroidal effusion in the left eye (**Figure 2J**). At the one and a half months follow-up, the patient’s condition remained stable, with visual acuity of 0.8 in the right eye and 0.6 in the left eye. OCT showed resolution of the serous retinal detachment in both eyes, with choroidal thickness returning to normal. In the right eye, the ellipsoid zone in the macular area appeared thinned, while the left eye showed disruption and loss of the ellipsoid zone, accompanied by high-reflective signals (**Figures 3G, H**).

## Literature review design

A search of the PubMed database was conducted for published cases of VKH-associated choroidal detachment. The keywords used were Vogt-Koyanagi-Harada disease/Vogt-Koyanagi-Harada Syndrome/Vogt-Koyanagi-Harada’s disease/choroidal detachment. Studies published in languages other than English were excluded. We reviewed the clinical features, multimodal imaging findings, treatments and prognosis of previously reported cases of choroidal detachment in VKH.

## Discussion

VKH is a multisystem autoimmune disease characterized by bilateral chronic granulomatous uveitis. Extraocular manifestations include neurological symptoms, auditory disturbances, as well as skin and hair abnormalities. Choroidal detachment (14), optic disc swelling type (15), and unilateral serous retinal detachment (16) are three uncommon clinical manifestations of VKH. This case is the first report of unilateral choroidal detachment in an elderly VKH patient, with multimodal imaging playing a crucial role in facilitating early diagnosis. FFA revealed severe optic disc leakage and peripheral retinal vessel wall staining, likely due to inflammation-induced vascular edema and leakage. The linear hypofluorescent areas in both eyes were attributed to the presence of choroidal folds, which were likely caused by choroidal thickening. When the patient first presented to our hospital, OCT revealed significant choroidal thickening of nearly 600 μm. The thickening was primarily associated with inflammatory infiltration and increased exudation (17). Elevated fluorescein leakage was observed in the temporal region of the left eye, indicating the presence of choroidal detachment. Severe choroidal inflammation was likely responsible for the choroidal detachment in this case. Inflammation can increase the permeability of the choroidal capillaries, resulting in vascular leakage (14, 18). The unilateral choroidal detachment in this patient occurred in the eye with more severe inflammation, and the rapid improvement following corticosteroid anti-inflammatory treatment further supports this cause. Jae et al. reported that compression of the vortex vein may lead to increased extravascular fluid retention and choroidal detachment (14). However, when our patient first presented to our hospital, she had a large number of inflammatory cells in the anterior chamber, posterior synechiae of the iris, significant cataracts, and vitreous opacities. Furthermore, due to the limitations of our equipment and imaging technique, both the 10-minute FFA and late-stage ICGA results were unclear, and we were unable to visualize the vortex vein. We recommend that future studies use ultra-widefield ICGA to further explore the mechanisms underlying choroidal detachment. Choroidal detachment is an uncommon manifestation of VKH, with only five cases of VKH-related choroidal detachment have been reported in English literature to date (14, 18–21). **Table 1** summarizes the clinical characteristics of these five cases. There were three males and two females, with an age of onset ranging from 59 to 68 years. The onset age of our patient was 76 years, suggesting a higher incidence of choroidal detachment in elderly VKH patients (22). Three of the patients were Japanese, while the other two were Caucasian and Korean, respectively, suggesting a higher prevalence of VKH-related choroidal detachment among Asians. All patients presented with bilateral choroidal detachment, with one patient additionally diagnosed with synovitis-acne-pustulosis-hyperostosis-osteitis syndrome (spondylitis and palmoplantar pustulosis). In four patients, choroidal detachment resolved following treatment with systemic corticosteroids alone or combined with immunosuppressants. The time required for treatment ranged from 4 to 95 days.

**Table 1:** The clinical features of previously reported cases of choroidal detachment in VKH.

Year	Gender	Age	Race	Choroidal detachment	Serous retinal detachment	Slit-lamp examination	FFA	ICGA	OCT	Extraocular manifestations	The time between onset of symptoms and the diagnosis of the CD	Prior anti-inflammatory treatment before the onset of the CD	Treatment	Prognosis
1996	Male	62	Japanese	bilateral	bilateral	In both eyes, the anterior chambers were shallow. A few cells and faint flare were observed.	FFA revealed many patches of hyperfluorescence at the level of the RPE in the arteriovenous phase and bilateral pooled fluorescein corresponding to the area of SRD in the late phase.	ND	ND	headache, nausea, and tinnitus	6 days	No	The patient was treated with pulse therapy of intravenous methylprednisolone (1,000 mg daily for 3 days) followed by tapering of oral prednisolone.	The SRD in the posterior fundus resolved gradually, and his BCVA was RE, 20/30 and LE, 20/200.
2004	Female	59	Japanese	bilateral	No	Both eyes showed normal sclera and a small number of cells in the anterior chamber. Fine KP were present, and the anterior chambers were slightly shallow. A few cells were detected in the vitreous.	FFA revealed alternating lines of hyperfluorescence and hypofluorescence that were created by the choroidal folds. There was severe leakage of dye from the optic disc and mild dye pooling in the subretinal space around the disc. Multiple mild leakages from peripheral retinal vessels over the detached choroid were also noted.	ND	ND	headache, nausea, and vomiting	5 days	ND	High doses of intravenous corticosteroids (betamethasone 20 mg/day) for 2 days. The systemic betamethasone was tapered to 8 mg/day in eight days, and a total of 112 mg of betamethasone was given. This was followed by oral prednisolone, 60 mg/day, which was also tapered.	Four days after the initiation of systemic corticosteroid therapy, the CD was completely resolved, and her corrected visual acuity was 1.0 in both eyes.
2017	Male	68	Caucasian	bilateral	bilateral	Granulomatous KP with significant shallowing of the anterior chambers, mild anterior chamber cell activity, and few areas of posterior synechiae.	FFA showed pooling of fluorescein in late frame in the subretinal space.	ICGA showed multiple areas of choroidal hypoperfusion.	OCT showed bilateral extensive subretinal fluid at the left macula and less so at the right.	mild headache	2-3 weeks	No	High-dose intravenous prednisolone (1 g of ntravenous methylprednisolone) for 3 days. Treatment was continued with a slowly tapering course of oral prednisolone.	Both CD and SRD were noted to resolve over a period of 1 week. Four weeks after his initial presentation, the visual acuit was 6/18 in the right eye and 6/36 in the left.
2018	Female	66	Japanese	bilateral	bilateral	Bilateral shallow anterior chambers. There were no KP in both corneas, and no cells in the anterior chambers.	FFA depicted multiple areas of pinpoint dye leakage from the RPE.	ICGA showed multiple areas of choroidal hypoperfusion in both eyes.	OCT showed ubretinal fluid with a wavy RPE line and thicker choroid in both eyes.	synovitis-acne-pustulosis- hyperostosis-osteitis syndrome (multiple spondylitis and palmoplantar pustulosis)	4 months	No	high-dose intravenous corticosteroids were given for 3 days. Tapering course of oral prednisolone to 30 mg per day for 8 days. Immunosuppressive medication of cyclosporine 150 mg twice daily.	Seventy days after the second course of high-dose intravenous corticosteroids, CD and SRD were resolved completely, and BCVA was 0.5 in the right eye and 0.7 in the left eye.
2021	Male	61	Korean	bilateral	No	Trace cells were observed in a slightly shallow anterior chamber.	On late-phase UWF FA, multiple dye leakage from RPE at the posterior pole and dye pooling at the posterior margin of CD were observed.	On UWF ICGA, patchy hyperpermeability and dilated veins drain to vortex, of which many were not visible due to CD, were noted in the late phase.	OCT of both eyes revealed multiple areas of subretinal fluids above a wavy thick choroid.	headache	1 week	No	He was treated with high doses of intravenous corticosteroid (methylprednisolone 1 g/day) for 3 days and a tapering dose of oral presdnisolone thereafter.	Four days after the initiation of systemic corticosteroid therapy, CD was completely resolved. At week 4, the visual acuity was 20/20 in the right eye and 20/25 in the left eye.

CD, choroidal detachment; SRD, serous retinal detachment; RPE, retinal pigment epithelium; KP, keratic precipitates; BCVA, best corrected visual acuity; FFA, fundus fluorescein angiography; ICGA, indocyanine green angiography; OCT, Optical coherence tomography; ND, no data.

Uveal effusion syndrome (UES) with peripheral choroidal detachment and exudative retinal detachment is an important differential diagnosis in this case. UES is characterized by ciliochoroidal detachment, exudative retinal detachment, and secondary RPE changes (23). UES can be classified into three types: Type 1 is characterized by nanophthalmos with scleral abnormalities, Type 2 involves normal axial length with scleral abnormalities, and Type 3 is idiopathic UES, presenting with both normal axial length and normal sclera (24). UES typically occurs in both eyes but usually presents without signs of anterior segment inflammation. Unlike the rapid onset of VKH, UES usually has a chronic onset and appears to be unresponsive to corticosteroid treatment (25). Our patient had normal axial length in both eyes, and ocular ultrasonography showed no evidence of scleral thickening. However, the rapid onset, presence of anterior segment inflammation, and good response to corticosteroid treatment help distinguish this case from idiopathic UES. Posterior scleritis is another important differential diagnosis in this case. It is an ocular inflammatory disease that typically presents with pain, redness, photophobia, and decreased vision. Around 40% of posterior scleritis cases are associated with systemic conditions, such as rheumatoid polyarthritis, systemic lupus erythematosus, and pANCA(+) systemic vasculitis (26). Ocular ultrasonography in posterior scleritis shows thickening of the choroid and sclera. A characteristic “T sign” can be observed when retrobulbar edema surrounds the optic nerve (27, 28). Posterior scleritis is typically unilateral, with only about one third of cases involving both eyes (29, 30). Our patient had no eye pain or the aforementioned ocular ultrasonography findings, and both of her eyes were affected simultaneously. Additionally, our case can be differentiated from choroidal metastasis. In later stages of choroidal metastasis, patients can exhibit pain, diplopia, uveitis, and secondary glaucoma due to metastasis to the ciliary body or iris. B-scan ultrasonography typically reveals an elevated subretinal mass and secondary exudative retinal detachment (31). OCT findings are characterized by the “lumpy bumpy appearance” (32). Our case did not show these imaging findings and can be differentiated through the patient’s medical history and systemic examination.

VKH presents with a wide range of clinical manifestations. In addition to choroidal detachment, the optic disc swelling type and unilateral exudative retinal detachment are also uncommon manifestations. The optic disc swelling type of VKH is primarily characterized by significant optic disc edema, with OCT detecting no or only minimal serous retinal detachment. Patients with optic disc swelling type VKH tend to be predominantly female and have an older age of onset. These patients usually have milder visual impairment prior to treatment; however, treatment is often delayed, increasing the risk of progressing to a chronic form of the disease (15). Nakao et al. suggested that the incidence of disc swelling is associated with disc morphology, with the cup-to-disc ratio in eyes with optic disc swelling in VKH was smaller (33). The main finding on FFA in this case was optic disc swelling. The patient’s age and optic disc morphology are consistent with the typical characteristics of the optic disc swelling type, but the presence of serous retinal detachment indicates a more severe inflammatory response in the left eye. If the patient presents with only optic disc swelling, differentiation from optic neuritis is necessary. EDI-OCT showed that the initial choroidal thickness and the changes in choroidal thickness before and after treatment are greater in the optic disc swelling type of VKH than in optic neuritis (34). The diagnostic criteria for VKH require involvement of both eyes (8). Although there are rare reports of unilateral VKH (16, 35–37), it is inherently a bilateral disease. EDI-OCT and ICGA can detect subclinical inflammation in the contralateral eye. Even with predominantly unilateral presentation, prompt diagnosis and systemic corticosteroid treatment are essential. Recognizing these atypical types of VKH is essential to help clinicians avoid missed diagnoses, misdiagnoses and treatment delay.

We report a case of an elderly female with VKH, presenting with unilateral choroidal detachment and bilateral serous retinal detachment, and describe her multimodal imaging findings. This case, along with previously reported cases, indicates that choroidal detachment is an uncommon manifestation of VKH. VKH-related choroidal detachment is more commonly seen in patients over 60 years old, typically presenting with optic disc swelling. Systemic corticosteroid therapy has proven to be effective in its treatment. Furthermore, multimodal imaging enables clinicians to make early and precise diagnoses of atypical VKH, leading to earlier detection, timely treatment, and improved outcomes.

## Data Availability

The original contributions presented in the study are included in the article/Supplementary Material. Further inquiries can be directed to the corresponding author.
